# The complete chloroplast genome of *Lycoris aurea* (L’Hér.) Herb

**DOI:** 10.1080/23802359.2020.1715296

**Published:** 2020-01-24

**Authors:** Ye Peng, Jing Wei, Limei Yang

**Affiliations:** Co-Innovation Center for Sustainable Forestry in Southern China, College of Biology and the Environment, Key Laboratory of State Forestry and Grassland Administration on Subtropical Forest Biodiversity Conservation, Nanjing Forestry University, Nanjing, China

**Keywords:** Chloroplast genome, *Lycoris aurea*, Amaryllidaceae, phylogenetic analysis

## Abstract

*Lycoris aurea* (L’Hér.) Herb is a herb widely growing in Chinese southen region, such as Guangxi, Guangdong, Fujian , Yunnan and Sichuan provinces. It not only has medicinal value, but also can be used as ornamental garden plant. The circular chloroplast genome of *L. aurea* was 158,690 bp in size, consisting of a pair of inverted repeat (IR) regions (26,782 bp) separated by a large single-copy (LSC) region (85,467 bp) and a small single-copy (SSC) region (18,541 bp) regions. And, it contained 127 genes, including 38 tRNA genes, 8 rRNA genes and 81 mRNA genes. The overall GC content of *L. aurea* is 37.73%. Phylogenetic analysis strongly supported that *L. aurea* and its congeneric species, *L. radiata* and *L. squamigera*, as sister group with 100% bootstrap value.

Amaryllidaceae is a family comprising several horticulturally important plant genera (Heywood et al. 2007). *Lycoris aurea* (L’Hér.) has been used in traditional Chinese medicine (TCM) for a long time. Phytochemical analysis showed that *L. aurea* contained various Amaryllidaceae alkaloids such as galanthamine, which can increase acetylcholine sensitivity and has a positive effect when treating Alzheimer’s disease as an inhibitor of cholinesterase (Dal-Bianco et al. 1991). Recently, an endophytic bacterium, isolated from *L. aurea*, is able to secrete a toxoflavin, which has fungicidal activity against human fungal pathogens (Li et al. 2019). Currently, the complete chloroplast (cp) genome for *Lycoris* was rarely reported except for the *L. squamigera* (Jin et al. 2018), *L. radiata*, *L. springer*, *L. longituba*(Zhang et al. 2019), none for *L. aurea*. Here, we report the complete chloroplast genome sequence of *L. aurea* to address this and to identify new regions of genomic variability.

This plant’s sample was taken from Du’an county, Guangxi Province, China (108°02′E, 24°06′N). The voucher specimen has been preserved at the Herbarium of Nanjing Forestry University (accession number Duan20120827012). The whole genome was sequenced using Illumina HiSeq2500 high-throughput Sequencing platform based on Sequencing By Synthesis (SBS) technology. A total of 20,826,789 clean reads were produced and then used for the *de novo* assembly with NOVOplasty 2.7.2 (Dierckxsens et al. 2016). The chloroplast sequence assembly results were annotated with gene structure under the use of CpGAVAS pipeline (Liu et al. 2012).

The circular chloroplast genome of *L. aurea* was 158,690 bp in size, consisting of a pair of IRs (26,782 bp) separated by LSC (85,467 bp) and SSC (18,541 bp) regions. And, it contained 127 genes, including 38 tRNA genes, 8 rRNA genes, and 81 mRNA genes. Among those, seven protein-coding genes (*rps19, rpl2, rpl23, ycf2, ndhB, rps7, rps12*), eight tRNA genes (*tRNA-CAA, tRNA-GUG, tRNA-CAU, tRNA-CAA, tRNA-GAC, tRNA-UUC, tRNA-UGC, tRNA-GUU*), and four rRNA genes (*rrn16, rrn23, rrn4.5 rrn5*) were duplicated within the IR regions. The overall GC content of *L. aurea* is 37.73%. Total number of identified SSRs is 51, and the number of repeats of single nucleotide, dinucleotide, and trinucleotide was 49,1, and 1, respectively. The annotated genomic sequence has been submitted to GenBank under the accession number MN831471.

To identify the phylogenetic position of *L. aurea* in Amaryllidaceae, we used MrBayes 3.2.6 (Ronquist et al. 2012) by downloading the chloroplast complete gene sequences of 16 species of Amaryllidaceae and 2 species of Asparagaceae from the NCBI to make phylogenetic tree (Figure 1). ModelFinder determined the best DNA substitution model (GTR + I + G + F). A Markov chain Monte Carlo (MCMC) was run for 100,000 generations and the initial 25% of sampled data were discarded as burn-in. The result showed that *L. aurea* and its congeneric species, *L. radiata* and *L. squamigera*, as sister group (posterior probability = 1.0; Figure 1).

**Figure 1.**
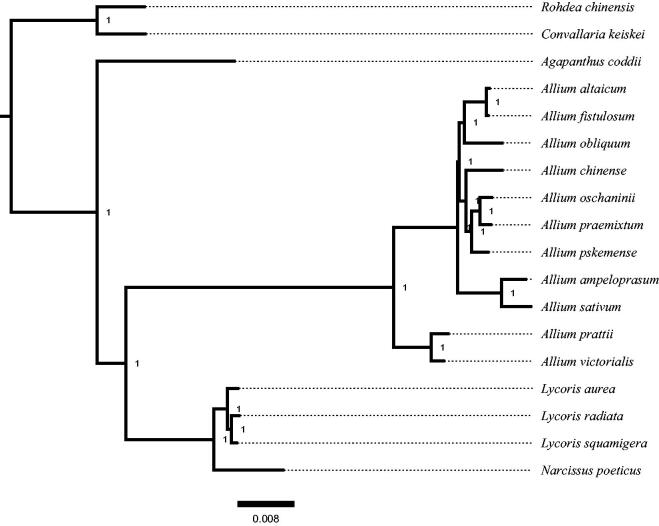
Phylogenetic relationships among 18 complete chloroplast genomes of Amaryllidaceae and Asparagaceae. Bootstrap support values are given at the nodes. Chloroplast genome accession number used in this phylogeny analysis: *Rohdea chinensi*: MH356725; *Convallaria keiskei*: NC042228; *Agapanthus coddii*: NC035971; *Allium altaicum*: NC040972; *Allium fistulosum*: NC040222; *Allium obliquum*: NC037199; *Allium chinense*: NC043922; *Allium oschaninii*: NC044470; *Allium praemixtum*: NC044412; *Allium pskemense*: NC044411; *Allium ampeloprasum*: NC044666; *Allium sativum*: NC031829; *Allium prattii*: NC037432; *Allium victorialis*: NC037240; *Lycoris aurea*: MN831471; *Lycoris radiata*: NC045077; *Lycoris squamigera*: NC040164; *Narcissus poeticus*: NC039825.
